# BCR-ABL1-Associated Reduction of Beta Catenin Antagonist Chibby1 in Chronic Myeloid Leukemia

**DOI:** 10.1371/journal.pone.0081425

**Published:** 2013-12-10

**Authors:** Elisa Leo, Manuela Mancini, Michela Aluigi, Simona Luatti, Fausto Castagnetti, Nicoletta Testoni, Simona Soverini, Maria Alessandra Santucci, Giovanni Martinelli

**Affiliations:** Istituto di Ematologia “Lorenzo e Ariosto Seràgnoli”, Dipartimento di Medicina Specialistica Diagnostica e Sperimentale - DIMES, University of Bologna - Medical School, Bologna, Italy; University of Miami School of Medicine, United States of America

## Abstract

Beta Catenin signaling is critical for the self-renewal of leukemic stem cells in chronic myeloid leukemia. It is driven by multiple events, enhancing beta catenin stability and promoting its transcriptional co-activating function. We investigated the impact of BCR-ABL1 on Chibby1, a beta catenin antagonist involved in cell differentiation and transformation. Relative proximity of the Chibby1 encoding gene (C22*orf*2) on chromosome 22q12 to the BCR breakpoint (22q11) lets assume its involvement in beta catenin activation in chronic myeloid leukemia as a consequence of deletions of distal BCR sequences encompassing one C22*orf*2 allele. Forty patients with chronic myeloid leukemia in chronic phase were analyzed for C22*orf*2 relocation and Chibby1 expression. Fluorescent in situ hybridization analyses established that the entire C22*orf*2 follows BCR regardless of chromosomes involved in the translocation. In differentiated hematopoietic progenitors (bone marrow mononuclear cell fractions) of 30/40 patients, the expression of Chibby1 protein was reduced below 50% of the reference value (peripheral blood mononuclear cell fractions of healthy persons). In such cell context, Chibby1 protein reduction is not dependent on C22*orf*2 transcriptional downmodulation; however, it is strictly dependent upon BCR-ABL1 expression because it was not observed at the moment of major molecular response under tyrosine kinase inhibitor therapy. Moreover, it was not correlated with the disease prognosis or response to therapy. Most importantly, a remarkable Chibby1 reduction was apparent in a putative BCR-ABL1+ leukemic stem cell compartment identified by a CD34+ phenotype compared to more differentiated hematopoietic progenitors. In CD34+ cells, Chibby1 reduction arises from transcriptional events and is driven by C22*orf*2 promoter hypermethylation. These results advance low Chibby1 expression associated with BCR-ABL1 as a component of beta catenin signaling in leukemic stem cells.

## Introduction

The BCR-ABL1 fusion gene is the causative genetic lesion of chronic myeloid leukemia (CML) [1]. It originates from t(9;22)(q34;q11) reciprocal translocation with the breakpoint on chromosome 9 falling within a >300 kb segment at the Abl 5′ end and the breakpoint on chromosome 22 within a 5.8 kb region spanning BCR exons 12–16 known as the major breakpoint cluster region (M-bcr). The resulting p210KDa chimeric protein has the ABL variable domain replaced by the first 902 or 927 amino acids of BCR, with the tetramer domain at the first exon-encoded N-terminus of BCR (encompassing amino acids 1 to 63) essential for converting inactive ABL into constitutively active BCR-ABL1 [2]. Accordingly, the majority of CML patients undergo complete hematologic remission in response to the tyrosine kinase (TK) inhibitor imatinib (IM) [3]. However, leukemic stem cells (LSC) are neither dependent on BCR-ABL1 TK activity for proliferation and survival nor killed by IM and second generation inhibitors nilotinib and dasatinib; therefore, they provide a sanctuary for the disease relapse upon drug withdrawal and a putative source of drug-resistance [4].

Beta Catenin is a central component of self-renewal of BCR-ABL1+ LSC and reprogramming of committed granulocyte/macrophage progenitors (GMP) into LSC at the blast crisis (BC) onset [5]–[7]. Moreover, it is involved in microenvironmental protection of CML stem and progenitor cells from TK inhibitors [8]. Multiple events contribute to beta catenin stabilization in CML. They encompass the BCR-ABL1-mediated beta catenin phosphorylation at specific tyrosine residues (Y86 and Y654), resulting in its impaired recruitment by the Axin/glycogen synthase kinase 3 beta (GSK3b) destruction complex, BCR-ABL1-associated overexpression of growth arrest specific 2 (GAS2), reducing its degradation by the calpaine system, and GSK3b inactivation due to the prevalence of a GSK3b mis-spliced isoform unable to phosphorylate beta catenin and/or to GSK3b de-phosphorylation by the Fas associated phosphatase 1 (Fap1) [9]–[12]. Subsequently, beta catenin enters the nucleus to form a transcription complex with TCF/LEF factors and activates the expression of target genes, such as c-Myc and cyclin D1 [13].

Chibby1 (Cby1) is a beta catenin antagonist encoded by C22*orf*2 on chromosome 22q12. Its nuclear interaction with the beta catenin C-terminal activation domain hampers beta catenin binding with TCF/LEF transcription factors, thereby repressing the target gene expression [14]. Moreover, Cby1 association with 14-3-3 scaffolding proteins ς and σ drives beta catenin nuclear export and cytoplasmatic relocation in a stable tripartite complex, attenuating beta catenin signaling [15]. Cby1 may, therefore, have a tumor suppressive function, and its down-regulation participate in cancer pathogenesis. Indeed, Cby1 downmodulation either due to C22*orf*2 loss or promoter hypermethylation is the most frequent genetic lesion in cranial pediatric ependymomas [16].

The relative proximity of C22*orf*2 to the BCR breakpoint on chromosome 22q11 suggest its putative involvement in beta catenin activation in CML. In particular, the loss of one C22*orf*2 allele as a consequence of deletions of distal BCR sequences occurring at the time of Philadelphia translocation may cause the gene haploinsufficiency eventually associated with disease worse prognosis [17]. Fluorescent in situ hybridization (FISH) analyses performed on bone marrow cells of forty CML patients in chronic phase (CP) showed that the entire C22*orf*2 follows BCR and relocates to the derivative chromosome 9 (der (9q)) in patients with the typical t(9;22)(q34;q11) translocation or to the second fusion gene in patients with variant translocations. Differentiated myeloid cells from bone marrow samples of thirty out of forty CML patients exhibited a reduction of Cby1 protein to less than half of reference values (healthy persons: HP), only in part dependent on transcriptional events. Cby1 reduction was not related to the disease risk according to Sokal score and with the response to TK inhibitors. At all instances, when present, it is a unique trait of clonal BCR-ABL1+ hematopoiesis because it was revoked at the moment of major molecular response (MMR) under TK inhibitor therapy. The most intriguing findings concern the significant reduction of Cby1 expression driven by the promoter hypermethylation in the putative LSC compartment identified by a CD34+ phenotype. The findings suggest that Cby1 is a component of beta catenin signaling, acting in concert with BCR-ABL1 to provide LSC an advantage over the normal counterpart.

## Materials and Methods

### Ethics Statement

CML patients included in the study provided their written informed consent to be enrolled in clinical trials NCT00769327, NCT01535391 and NCT01061177 (ref clinicaltrials.gov) and to be evaluated for response to therapy with imatinib or nilotinib according to the study design. The above mentioned clinical trials were approved by the Ethical Committee of the Policlinico S.Orsola-Malpighi on July 15th 2008, September 13rd 2011 and June 29th 2010, respectively. Patients gave the written consent to the use of their bone marrow samples for genetic and laboratory biomarker analyses. Moreover, they gave verbal informed consent to participate in this particular study at the moment of their enrollment in the clinical trials, according to the guidelines of the Ethical Committee of the Policlinico S.Orsola-Malpighi. The verbal informed consent was properly mentioned in the medical record. Once collected, all samples were provided with an anonymous code number and stored following the recommendations of aforementioned trails. The samples from 8 healthy subjects were collected at the moment of hematopoietic stem cell harvest from peripheral blood following mobilization and intended for bone marrow transplantation. Participants were verbally informed and consented to the use of harvested sample residual fraction for this study. The verbal consent was registered in their medical records according to the consent procedures approved by the Ethical Committee of the Policlinico S.Orsola- Malpighi. All methods employed in this study were in accordance with the Declaration of Helsinki.

### Study Population

Forty CML-CP patients were included in our study. Clinical details are given in Table S1. In brief, all of them exhibited the BCR-ABL1 rearranged gene coding for p210 fusion protein and were treated with TK inhibitors (imatinib or nilotinib). Thirty two patients achieved a complete hematological response (CHR). All but one (35 of Table S1) achieved a MMR (3 log reduction of BCR-ABL1 transcript level compared with that at diagnosis) within the 6th month or 1st year of therapy. The remaining eight patients had an insufficient follow-up for the evaluation of molecular response. Five patients were compared for Cby1 expression at diagnosis and at the moment of MMR. In 6 patients, Cby1 expression in mononuclear cell fractions (MCF), almost completely composed of differentiated myeloid progenitors, was compared with that of the putative LSC compartment identified by a CD34+ phenotype. Informed consent to report clinical details of patients and results of biomolecular analyses was preliminarily obtained according to protocols NCT00769327, NCT01535391, and NCT0161177 (see the above section for details).

### Selection of Cells

Bone marrow samples from CML-CP patients and peripheral blood samples from HP were purified by Ficoll-Hypaque (Cederlane) density gradient centrifugation (1,000 g for 30′) to isolate MCF containing myeloid progenitors and more mature cells from red cells and plasma. Immunomagnetic selection (mini-MACS from Miltenyi Biotec) was used to purify CD34+ cells from MCF according to published method [18]. In brief, MCFs (1–5×10^8^/mL) were incubated at 4°C for 15′ with magnetic microbeads coated with anti-CD34 antibody (Miltenyi Biotec). Cell suspension was thereafter applied to a separation column placed in a magnetic field, which retains magnetically stained cells. After elution, cells were counted and assayed for their viability by the Trypan blue exclusion test. The recovery of CD34+ cells from MCF of CML-CP and HP was 0.29±0.11% and 0.17±0.03%, respectively. Cell purity was confirmed using flow cytometric analysis of anti-CD34-FITC antibody (BD Biosciences); it was >90% in all cases (data not shown). Cytogenetic analysis confirmed previous findings, revealing the Philadelphia (Ph1) chromosome in 96.5±1.2% of CD34+ cell from CML-CP patients (data not shown).

### Fluorescent in-situ Hybridization (FISH)

Dual color FISH for C22*orf*2 was performed on fixed metaphases using two different probes (RPI-199H16 22q12 from 37239655 to 37325154 and RPI-172B20 22q12 from 38345395 to 38559115 from Technogenetics). In brief, once placed on slides, probes were co-denatured at 75°C for 5 minutes and hybridized at 37°C overnight using Hybrite (Vysis). After washing in SSC 0.4X at 72°C for 2′ and SSC 2X+0.05% Igepal at room temperature for 30″, the slides were counterstained with 4′,6-diamidino-2-phenylindole (DAPI) and analyzed under a fluorescent microscope equipped with FITC/TRITC/AQUA/DAPI filter sets and Genikon imaging system software (Nikon Instruments). The LSI BCR/ABL tri-colour dual-fusion (DCDF) translocation probe (Vysis) was used to detect t(9;22)(q34;q11) translocation in interphase or metaphase nuclei [19]. All images were acquired using a 100× objective.

### RNA and Protein Analysis

A commercial kit (SV total RNA Isolation System, Promega) was used for total RNA extraction starting from 1×10^6^ cells. RNA was converted in cDNA using ImProm-II Reverse Transcription System (Promega) in a 50 µl final-volume reaction mix comprising 2 µg of total RNA, 10 µl of reaction buffer (1×), 3 mM Mg2+, 0.5 mM dNTPs each, 0.5 µg of random hexamers, 1 U Recombinant RNasin Ribonuclease Inhibitor, and 160 U ImProm-II Reverse Transcriptase. The reverse transcription reaction was performed using the following program: 25°C for 5′, 40°C for 80′, and 75°C for 15′. PCR amplifications were performed with 1.25 U of Taq DNA Polymerase kit (Roche) in 30 µl of reaction buffer containing 0.4 µM of each primer, 0.2 mM dNTPs, and 500 ng of RT product. Thirty-two amplification cycles were performed after a 4′ denaturation step at 95°C, followed by a denaturation step at 95°C for 30″, a primer annealing step at 59°C (Cby1 and beta-2-microglobulin (B2M)) or 58°C (cyclin D1), and an elongation step at 72°C for 30″. The following primers were used: 5′- AGAGTCCTTGCTGGGGGTTCG-3′ (upper) and 5′- CTCCACCTCCCGGGTTGATCG-3′ (lower) to amplify the two isoforms (200 and 340 bp) of Cby1, 5′-CCGCAATGACCCCGCACGAT-3′ (upper) and 5′-GCCTGGCGCCCTCAGATGTC-3′ (lower) to amplify cyclin D1 (442 bp), and 5′-CTCGCGCTACTCTCTCTTTCT-3′ (upper) and 5′- TCACATGGTTCACACGGCAGGC-3′ (lower) for to amplify B2M (289 bp) as control for RT efficiency. The amplification products were resolved in 2% agarose gel, and signal intensities were measured using a dedicated software (IMAGEJ 1.44 p Launcher software from National Institutes of Health, Bethesda, MD, USA). Protein expression in whole cell lysates of MCF and CD34+ cells was evaluated using Western blot (WB) according to standard methods using a Cby1 antibody kindly purchased by K.I. Takemaru [20]. To avoid individual differences in Cby1 expression, equal amounts of RNA and proteins from peripheral blood of 8 HP were pooled. The RNA and protein pool from HP was used in all experiments as control for PCR and WB from CML-CP patients. No differences in PCR and WB signal intensities obtained in 3–4 preliminary experiments, conducted in individual HP samples, did not exceed 10%. Preliminary experiments were conducted to exclude differences in Cby1 expression relative to the cell source, either bone marrow or peripheral blood (data not shown).

### Cby1 Promoter Methylation Status

MethylCollector Ultra Kit (Active Motif) was used to enrich the methylated DNA. In brief, 4 µg of total purified DNA were digested for 2 h at 37°C by 10 U of MseI, a methylation insensitive restriction enzyme. A total of 500 ng of fragmented DNA were processed under low salt-binding conditions according to the manufacturer’s instructions to obtain DNA enriched in methylated CpG islands, which was amplified using 0.4 µM of each primer encompassing region −85 to +120 of CBY1 promoter (5′- AGGTCAGTGATCCAGCTGCTTGT-3′ and 5′- ACTCATGCTGCACACCCGGC-3′). The following PCR conditions were used: initial denaturation at 95°C for 10′, 35 cycles (95°C for 30″, 58.5°C for 30″ and 72°C for 30″), and a final extension at 72°C for 4′. Input DNA was kept as internal control for PCR after the isolation of 5 mC-enriched DNA.

### Statistical Analysis

Signal intensities of PCR amplification products and WB obtained in at least three separate experiments were quantified by a dedicated software (IMAGEJ 1.44 p Launcher software from National Institutes of Health, Bethesda, MD, USA). In brief, the bands corresponding to PCR products and proteins in single gels and blots were selected and evaluated for their intensity by the aforementioned software. Histograms indicating the pixel intensity and area of each band were subsequently elaborated to give the mean values and standard deviations from at least three separate experiments. Those of pooled RNA and protein samples from HP were used as the reference (corresponding to 1) for *C22orf2* transcript and Cby1 protein levels. Student’s *t* test was used to evaluate the statistical significance of differences in signal intensities of PCR and WB analyses of CML-CP vs HP samples. P values <0.05 are considered statistically significant.

## Results

### C22*orf2*, the Cby1-encoding Gene, follows BCR Sequences and Relocates to the der(9q) Chromosome

**Figure 1:**
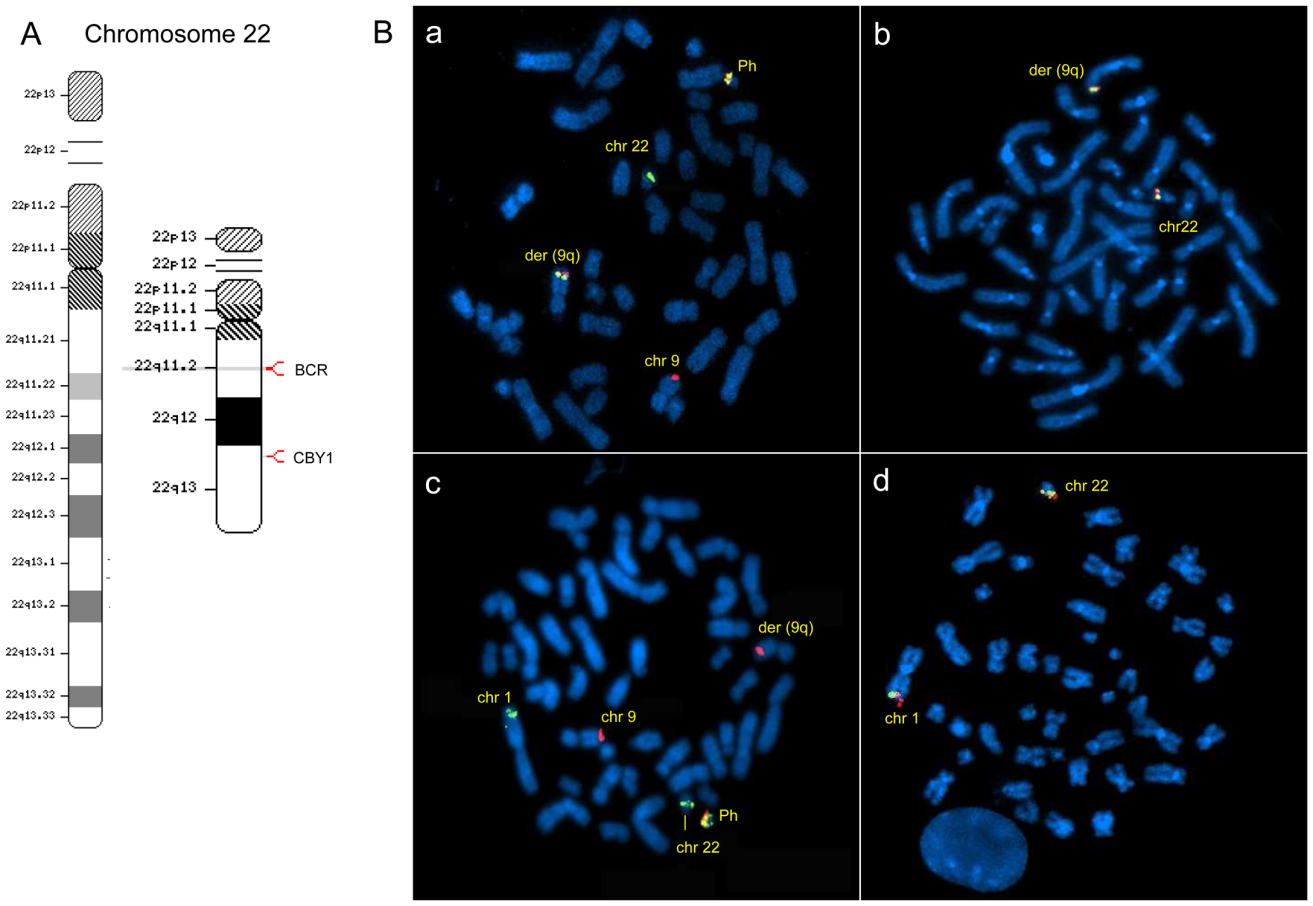
*C22orf2*, the Cby1-encoding gene, follows BCR sequences and relocates to the der(9q) or third chromosome involved in variant translocations. **A-** Location of BCR and Cby1 genes on chromosome 22 are shown with red marks on chromosome 22 ideogram using NCBI Map Viewer (http://www.ncbi.nlm.nih.gov/mapview/). **B- panel a:** typical FISH pattern of BCR-ABL1 rearrangement with one fusion signal at der(22q), one green signal at the non-rearranged 22q, and one red signal at 9q in the metaphases of a CP-CML patient with t(9;22) translocation; **panel b:** one pair of Cby1 signals, with the green one labeling the promoter origin and the red one the end of the gene, was translocated to der (9q) in metaphases of a CML-CP patient with t(9;22) translocation; **panel c:** the green signal corresponding to BCR was relocated to chromosome 1 in one patient exhibiting the t(1;9;22) variant translocation (#9 of Table S1); **panel d:** in the last case, Cby1 signals were relocated at the third chromosome involved in translocation. The results shown in panel b were confirmed in CML-CP patients with t(9;22) translocation included in the study, and those shown in panel d were confirmed in two CML-CP patients with variant translocations encompassing chromosomes 1 and 7 (see Figure S2). All images were acquired using a 100× objective. FISH analyses were performed according to procedures illustrated in the Materials and Methods section. At least 20 metaphases were analyzed for the detection of BCR-ABL1 and C22orf2 signals.

In 10%–18% of CML patients, the t(9;22) reciprocal translocation, which generates the BCR-ABL1 rearranged gene, is an unbalanced lesion where genomic sequences of either chromosome 9 or 22 may get lost [17]. Such deletions are adjacent to the t(9;22) breakpoint and may span several megabases of chromosomes 9 and 22. Several lines of evidence suggest that they are early events occurring at the time of translocation and producing a genetic heterogeneity *ab initio* associated with the disease prognosis [21]. The relative proximity of Cby1-encoding gene *C22orf2* (22q12) to the BCR breakpoint (22q11) suggests its BCR-ABL1-associated deletion as a putative component of beta catenin activation in CML cells (Figure 1A). FISH patterns of BCR/ABL1 and C22*orf*2 were investigated in MCF from bone marrow samples of CML-CP patients (see Table S1 for clinical details). FISH pattern in MCF from HP peripheral blood consists of two green and red signals, marking ABL on chromosomes 9 and BCR on chromosomes 22, respectively (Figure S1, panel A) All CML-CP patients with typical t(9;22) translocation displayed one BCR-ABL1 fusion signal at the Ph1 chromosome (22q-), one green signal corresponding to normal BCR at 22q and one red signal corresponding to normal ABL at the 9q (Figure 1B-panel A). The green signal corresponding to the BCR sequence was relocated to chromosome 1 in one patient exhibiting the t(1;9;22) variant translocation (Figure 1B-panel C relative to patient 9 of Table S1). BCR relocation at a third chromosome was confirmed in the other two CML patients with variant translocations (patients 3 and 27 of Table S1, data not shown). The C22*orf*2 probe used for FISH analyses encompasses the whole gene length with a green signal at the promoter origin and a red signal at the gene end (Figure S1-panel B). In CML-CP patients with typical t(9;22) translocation, one pair of C22*orf*2 signals was translocated to der(9q) chromosome (Figure 1B-panel B). C22*orf*2 signals were located at the third chromosome in patients with variant translocations (Figure 1B-panel D relative to patient 9 of Table S1 and Figure S2 showing FISH patterns of two additional patients with t(7;9;22) and t(1;9;22) translocations not included in the study). The findings support that the C22*orf*2 allele located on the chromosome 22 involved in t(9;22) translocation follows BCR sequences regardless of the type of translocation.

### Leukemic Myeloid Progenitors Exhibit a Prominent Reduction of Cby1 Protein Associated with BCR-ABL1

**Figure 2:**
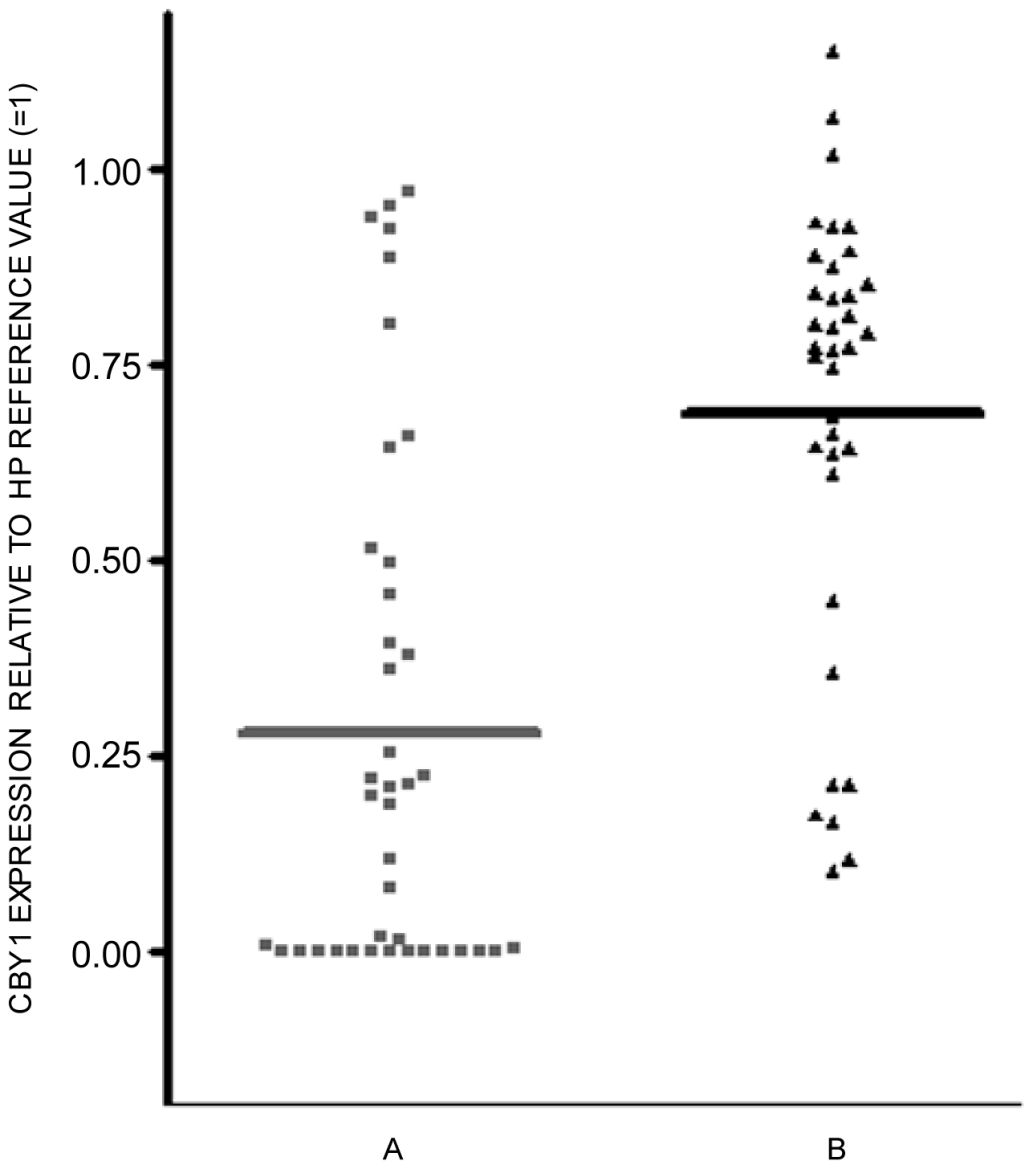
Cby1 reduced expression associated with BCR-ABL1. Cby1 protein was reduced below 50% HP in 30/40 CML-CP patients included in our study (mean: 0.278). Cby1 transcript reduction below the aforesaid value was only seen in 8/37 patients (mean: 0.671). The scattered distribution of points suggests individual differences in Cby1 expression in CML-CP patients compared with the HP group. The expression levels of Cby1 protein and transcript were assayed in MCF from bone marrow samples of CML-CP patients using WB and PCR. Single points are the median values of three separate experiment, with standard deviation not exceeding 10% (data not shown). Signal intensities of WB and PCR obtained with equal amounts of proteins and RNAs from MCF of HP peripheral blood samples pooled to avoid individual differences in gene expression were used as reference values ( = 1). Further details on the procedure used for signal intensity quantification were given in the Materials and Methods section.

**Figure 3:**
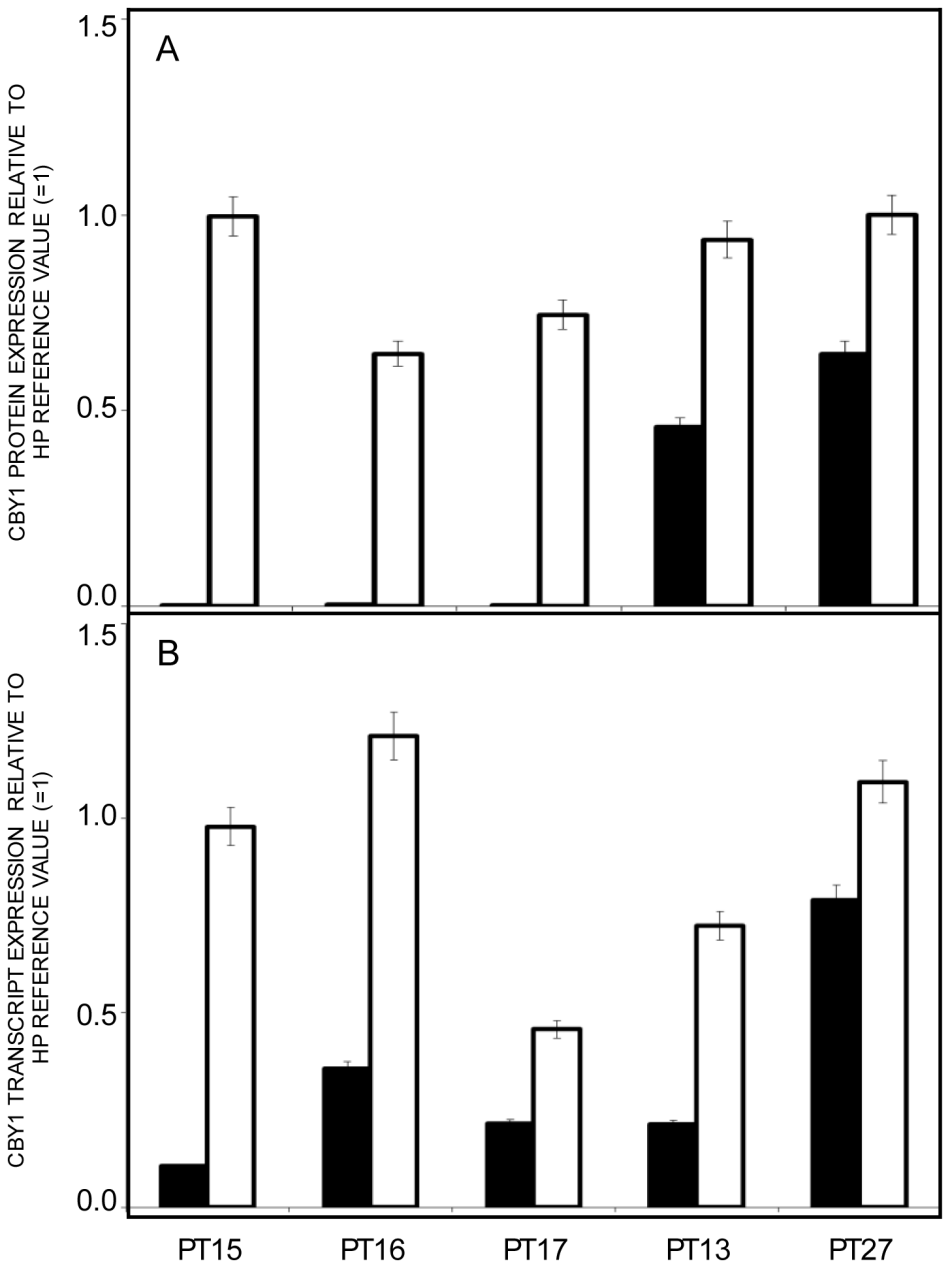
Cby1 reduced expression in MCF of CML-CP patients is restricted to the leukemic clone. At the moment of MMR Cby1 protein (**A**) and transcript (**B**) were significantly increased in all cases (p<0.05 or less) and approached the HP levels in 3 cases (see Table S3 for signal intensity details and Figure S2 for blots). The levels of Cby1 protein and transcript were evaluated in MCF from bone marrow samples of 5 CML-CP patients at diagnosis (black columns) and at the moment of MMR under TK inhibitor therapy (white columns). At this instance, a 3-log reduction in BCR-ABL1 transcripts compared to diagnosis was seen (data not shown). Cby1 expression was expressed as described in the legend to Figure 2, with HP signal intensities used as reference ( = 1).

Cby1 downmodulation has a role in the sustained activation of Wnt/beta catenin signaling in pediatric ependymomas and colon cancer cell lines [16], [22]. Cby1-enforced expression in the latter cell context accordingly induces beta catenin inactivation by promoting its nuclear export [23]. The crucial role of beta catenin in CML pathogenesis and progression prompted us to investigate the BCR-ABL1 impact on Cby1 expression [5], [24]. Bone marrow MCF of the majority of CML-CP patients (30/40) included in our study exhibited a reduction of Cby1 protein below 50% of the reference value (corresponding to the WB signal intensity of equal amounts of proteins from HP peripheral blood pooled to avoid individual differences) with a median value of 0.278 (Figure 2A-left panel and Table S2). In this cell context, an equivalent decrease of Cby1 transcript was apparent in a minority of patients (8/37), with a median value of 0.671 (Figure 2B-right panel and Table S2). In all but one (patient 22 of Table S1), the reduction of Cby1 transcript below 50% of the reference value was associated with low protein levels (-Table S2). The findings suggest that C22*orf*2 transcriptional downmodulation in more differentiated hematopoietic progenitors of CML-CP has a marginal impact on Cby1 protein expression, whose levels are probably regulated by complementary events affecting the protein stability. The expression levels of Cby1 transcript and/or protein were not correlated with the disease prognosis according to the Sokal score (Tables S1 and S2). At all instances, Cby1 reduction was strictly dependent upon BCR-ABL1 expression. Bone marrow MCF of five CML-CP patients with different Cby1 expression levels at diagnosis exhibited a significant increment of Cby1 protein and transcript at the moment of MMR (p<0.05 or less) (Figures 3A and B, Figure S3 and Table S3). These results let conclude that Cby1 reduced expression is an inherent trait of clonal BCR-ABL1+ hematopoiesis, only partly dependent upon transcriptional events and not correlated with the disease prognosis.

### Cby1 Downmodulation in the BCR-ABL1+/CD34+ LSC Compartment is Associated with Beta Catenin Nuclear Location and Transcriptional Activation

**Figure 4:**
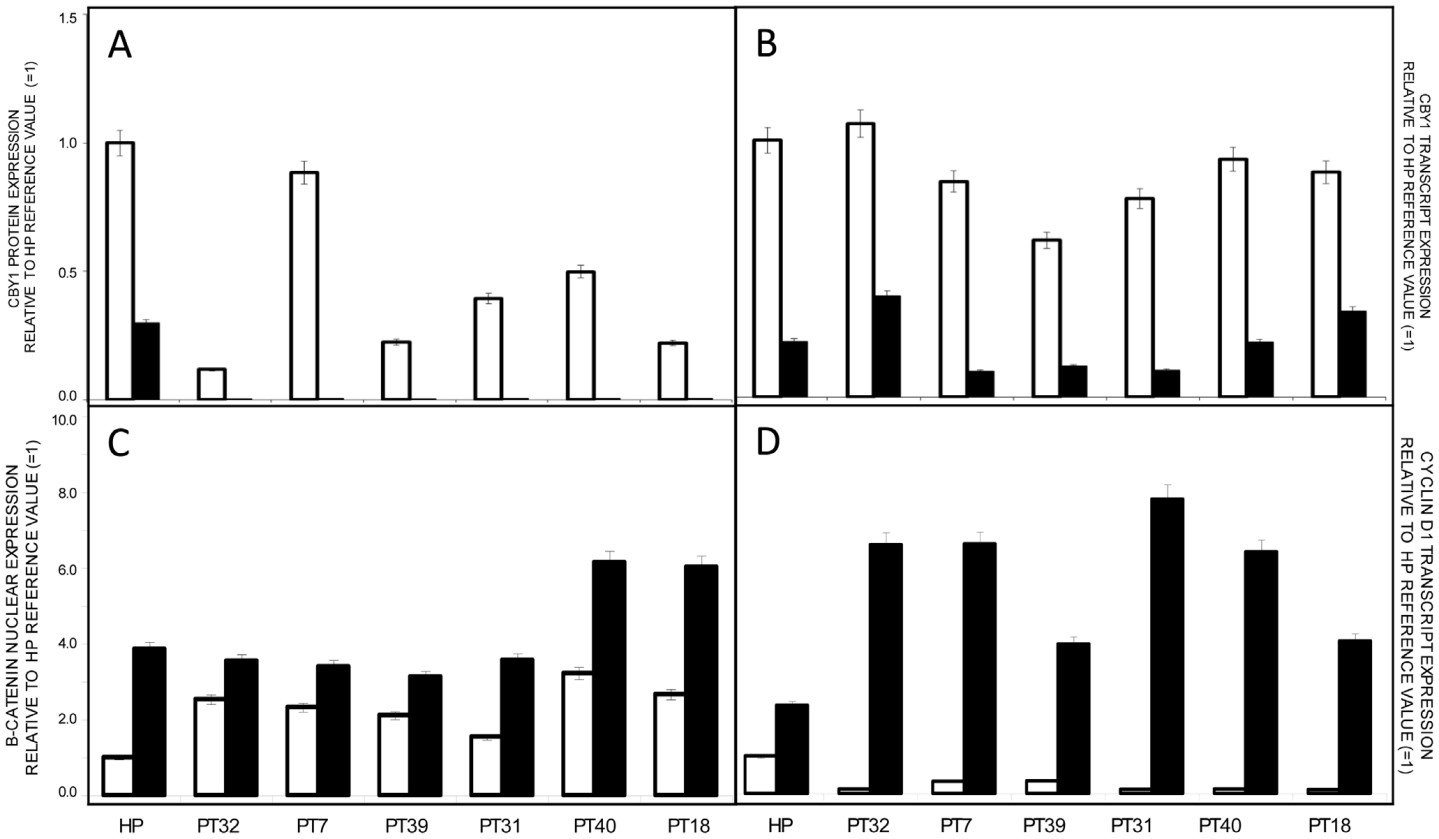
Prominent reduction of Cby1 expression in the putative LSC compartment identified by a CD34+ phenotype. The expression of Cby1 protein (**A**) and transcript (**B**) was significantly reduced (p<0.001 or less) in bone marrow CD34+ early progenitors of 6 CML-CP patients at diagnosis (black columns) compared with MCF (white columns). Cby1 reduction in CD34+ cells was associated with a significant increment of nuclear beta catenin protein (**C**) and cyclin D1 transcript (**D**) (p<0.05 or less). Cby1 reduction and nuclear beta catenin and cyclin D1 increments were also seen in CD34+ cell from HP. As in Figures 3 and 4, WB and PCR signal intensities of RNA and protein pools of HP were normalized to 1 and kept as reference of Cby1 expression in either cell type (see Table S4 for details on ratios and Figure S4 for blot images).

The central role of Wnt/beta catenin signaling in CML LSC proliferation and persistence under TK inhibitor therapy suggests a differentiation-dependent regulation of its antagonists, including Cby1 [5], [7]. Bone marrow MCF from six CML-CP patients exhibiting levels of Cby1 transcript similar or equal to those of HP pool were compared for Cby1 expression in bone marrow MCF and the putative LSC compartment identified by a CD34+ phenotype. The CD34+ compartment was chosen because it almost completely consists of BCR-ABL1+ cells (97±2%) still retaining a self-renewal potential, while the more immature Lin−/CD34− compartment mostly encompasses the residual normal hematopoietic stem cells (HSC) (78±2%) [18]. Cby1 transcript levels in CD34+ cells from all six CML-CP patients were significantly lower compared with MCF and protein expression was further reduced (p<0.001 or less) (Figures 4A and B, Figure S4 and Table S4). Notably, Cby1 expression was also significantly reduced in CD34+ cells from HP (p<0.001), supporting the participation of Cby1 downmodulation in beta catenin signaling in normal HSC (Figures 4A and B, Figure S4 and Table S4) [25]–[27]. Cby1 interaction with 14-3-3 scaffolding proteins ς and σ (in a stable tripartite complex encompassing beta catenin and driving beta catenin nuclear export) intervenes in the attenuation of beta catenin signaling [15]. Accordingly, Cby1 reduced expression in CD34+ cells of CML-CP patients and HP was associated with a significant increment of nuclear beta catenin and enhanced transcription of cyclin D1, a Wnt/beta catenin target gene involved in the maintenance of CD34+ pool (p<0.05 or less) [28]. These findings confirm and expand the results of a recently published study, showing that Cby1-enforced expression is a central component of beta catenin nuclear export and suppression of its transcriptional activity in BCR-ABL1+ cells [29]. In particular, they emphasize Cby1 participation in beta catenin signaling in the LSC compartment responsible for the disease pathogenesis and persistence under TK inhibitor therapy.

### Cby1 Transcriptional Downmodulation in CD34+ Cells is Driven by C22*orf2* Promoter Hypermethylation

**Figure 5:**
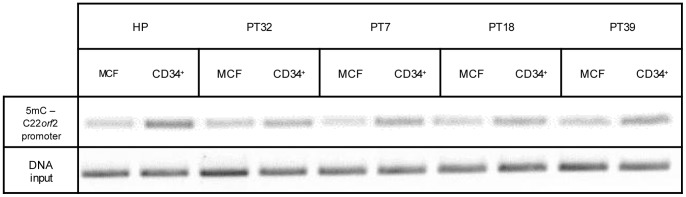
Cby1 reduced transcription in CD34+ cells is driven by DNA hypermethylation of C22*orf*2 promoter. PCR amplification of methylated DNA lets detect a significant increment of 5*orf*2 promoter encompassing nucleotides −85 to +120 in CD34+ cells of HP and 4 CML-CP patients compared to MCF (p<0.05 or less). The DNA input after the isolation of 5 mC-enriched DNA was used as an internal control for PCR.

The hypermethylation at DNA promoter associated CpG islands is a common mechanism of putative tumor suppressor gene transcriptional silence associated with BCR-ABL1 at some instances associated with CML progression and/or IM resistance [30]–[42]. Moreover, it is involved in the almost complete loss of protein tyrosine phosphatase receptor type γ (PTPRG), which causes the persistent activation of BCR-ABL1 TK [43]. Notably, DNA hypermethylation plays a central role in HSC protection from the activation of differentiation programs and is an epigenetic trait of a greater number of tumor suppressor genes in BCR-ABL1+/CD34+ compared with more differentiated progenitors [44], [45]. MCF and CD34+ cells from four CML-CP patients, previously investigated for Cby1 expression, and HP were, therefore, compared for 5-methyl cytosine (5 mC) content at a C22*orf*2 promoter region encompassing the region −85 to +120. As expected, leukemic CD34+ cells displayed significantly higher amounts of 5 mC at the aforesaid gene promoter region (p<0.01 or less) (Figure 5). The 5 mC excess was also apparent in CD34+ cells from HP, supporting the role of hypermethylation in lowering Cby1 expression, a central component of beta catenin signaling both in HSC and LSC.

## Discussion

Beta Catenin has a central role in the maintenance of CML LSC and BCR-ABL1 leukemogenesis [5], [7], [8]. Its aberrant signaling in leukemic cells is mostly dependent on multiple mechanisms enhancing the protein stability [9]–[12]. Firstly, BCR-ABL1-induced phosphorylation of beta catenin at specific tyrosine residues (Y86 and Y654) required for binding to the TCF4 transcription factor and transactivating function prevents its recruitment by the Axin/GSK3 complex thereby impairing its ubiquitination and proteasome degradation [9]. FAP1-dependent inactivation of GSK3 and the resulting block of beta catenin inhibitory phosphorylation at serine/threonine residues is a further component of BCR-ABL1-associated reduction of beta catenin degradation [12]. Moreover, the BCR-ABL1-dependent increase of GAS2, whose overexpression has been linked with CML progression, impairs the alternative route to beta catenin degradation by calpain [11], [46]. FAP1 and GAS2 are both targets of the interferon consensus sequence binding protein (ICSBP), whose expression is reduced in CML [47]. Finally, BCR-ABL1 recruitment and activation of JAK2 enhances beta catenin stability and activity and induces SET-mediated functional inactivation of protein phosphatase 2A (PP2A) which, in turn, promotes beta catenin activation by impairing GSK3 phosphorylation [48], [49]. At all instances, nuclear import is the essential prerequisite of beta catenin transcriptional activation [13]. Beta Catenin shuttling between cytoplasmic and nuclear compartments is regulated by multiple mechanisms encompassing posttranscriptional modifications (phosphorylation, acetylation and glycosylation) and transporters either promoting protein nuclear import or export [29]. Cby1 may be included in the class of beta catenin carriers because it relocates beta catenin to the cytoplasm in a tripartite complex encompassing 14-3-3 [15]. Accordingly, Cby1 enforced expression in colon cancer and CML cell lines promotes beta catenin nuclear export and transcriptional attenuation [23], [29]. The salient result of our study concerns Cby1 downmodulation associated with BCR-ABL1+ as a component of beta catenin activation in LSC. Cby1 downmodulation is an intrinsic trait of more differentiated leukemic cells (bone marrow MCF) (Figures 2 and 3, Tables S2 and S3 and Figure S3). It is not caused by gene haploinsufficiency due to deletions of distal BCR sequences involved in translocation (Figure 1, Figure S2). Moreover, it is only partly dependent upon transcriptional events (Figure 2, Table S2). Further investigation is in progress to elucidate the mechanisms involved in BCR-ABL1-associated reduced stability of Cby1 protein. Interestingly, Cby1 expression in more differentiated leukemic progenitors and mature myeloid precursors was not correlated with the disease prognosis or response to therapy, suggesting that in such cell compartments the impact of Cby1 expression on beta catenin signaling may be compensated by complementary signals affecting beta catenin stability (Tables S1 and S2) [9], [11], [12], [48].

**Figure 6:**
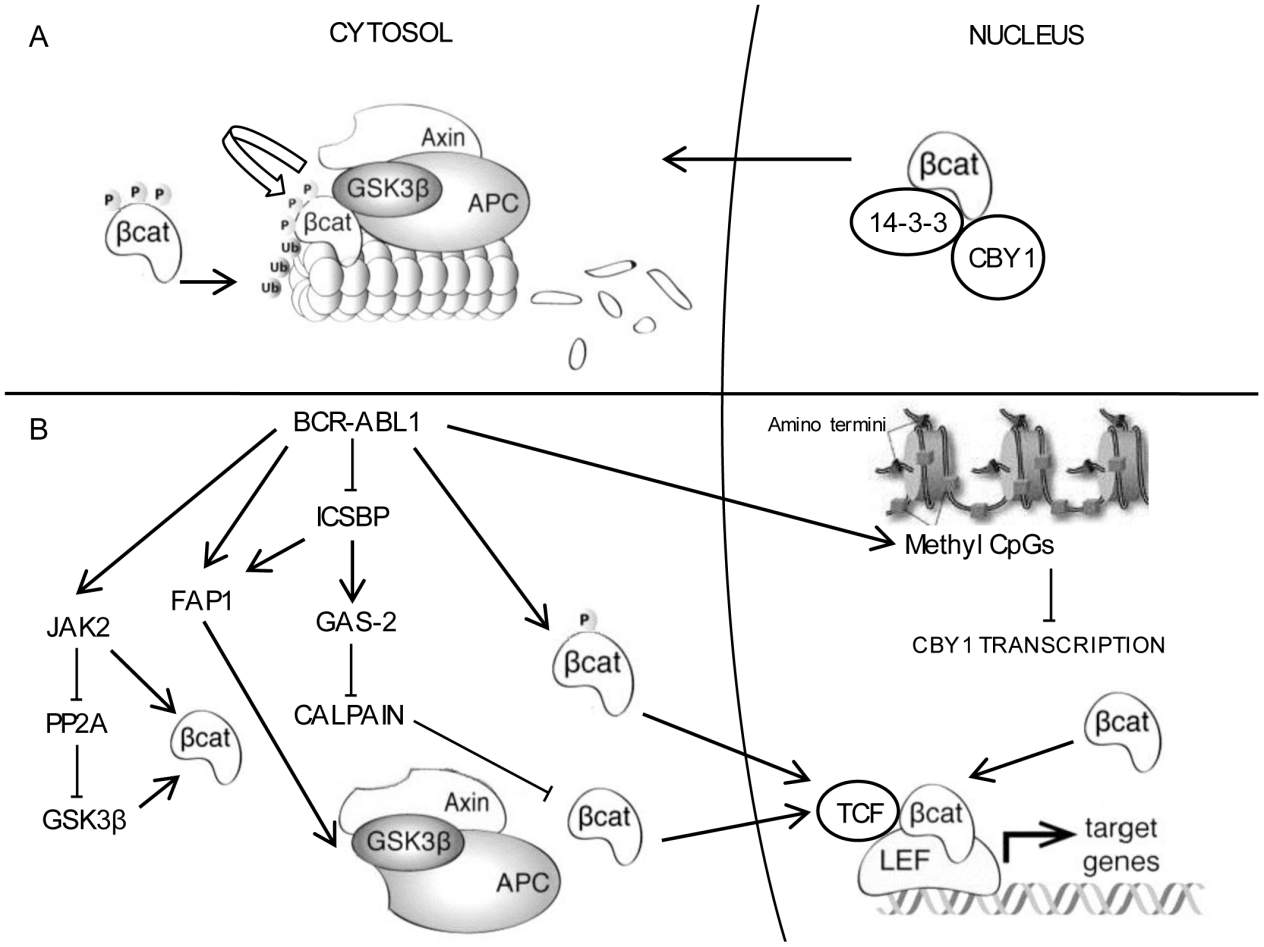
A graphic scheme showing that Cby1 is one component of beta catenin activation associated with BCR-ABL1. **A -** In unstimulated cells, beta catenin is exported from the nucleus into the cytoplasm by Cby1 in a tripartite complex encompassing 14-3-3 ξ and ο. Cytoplasmic beta catenin is subsequently phosphorylated at serine residues (41, 37 and 33) by GSK3 in a complex referred to as the destruction complex encompassing GSK3, Axin, and adenomatous polyposis coli (APC) and targeted for ubiquitination and proteasomal degradation [13]. **B**: In CML LSC, BCR-ABL1-driven promoter methylation induces Cby1 transcriptional downmodulation, which contributes to beta catenin nuclear retention and signaling. The other components of beta catenin signaling in CML cells include the BCR-ABL1 TK-driven phosphorylation of beta catenin at tyrosine residues (86 and 654), which precludes its recruitment at the destruction complex, and interferon consensus sequence binding protein (ICSBP)-dependent mechanisms, which through the increase of FAP1 phosphatase (which dephosphorylates and inactivates GSK3) and GAS2 calpain inhibitor, decreases beta catenin degradation by proteasome and calpain [9], [11], [12]. Finally, JAK2 activation enhances beta catenin activity and induces SET-mediated inactivation of PP2A thereby precluding PP2A-induced activation of GSK3 [49].

Cby1 downmodulation in BCR-ABL1+/CD34+ cells was correlated with beta catenin nuclear location and transcriptional activity (Figure 4 and Table S4 and Figure S4). Although CML CD34+ cells are not the earliest LSC, they retain an adequate self-renewal potential to reconstitute leukemic hematopoiesis in animal models and, more importantly, they constitute a homogeneous compartment harbouring the BCR-ABL1 gene [18]. In such cell context, Cby1 lower expression compared with more differentiated myeloid progenitors arises from transcriptional events driven by DNA hypermethylation at the gene promoter (Figures 4 and 5, Table S4 and Figure S4). Notably, the hypermethylation at DNA promoter-associated CpG islands is a common mechanism of tumor suppressor gene silence in CML at some instances associated with the disease progression and/or IM resistance [30], [32], [39], [45]. It is worth noting that Cby1 transcriptional downmodulation proceeding from promoter hypermethylation was also apparent in CD34+ cell from HP, supporting the central role of Cby1 in beta catenin signaling of HSC (Figure 4 and 5, Table S4 and Figure S3) [44]. In CML, Cby1 downmodulation complements BCR-ABL1-dependent events promoting beta catenin stabilization and nuclear import which provide LSC an advantage over the normal counterpart (Figure 6).

## Conclusions

Our study provides evidence of reduced expression of the beta catenin antagonist, Cby1, associated with the BCR-ABL1 rearranged gene of CML. Cby1 reduction in a cell context encompassing more differentiated hematopoietic progenitors (bone marrow MCF) is neither dependent upon gene haploinsufficiency due to deletions of distal BCR sequences nor correlated with the disease stage at diagnosis or response to TK inhibitors. It most likely encompasses post-transcriptional events affecting the protein stability. Notably, Cby1 expression was remarkably reduced in the putative BCR-ABL1+ LSC compartment identified by a CD34+ phenotype compared with differentiated leukemic progenitors. In this cell context, Cby1 downmodulation was evoked by transcriptional events driven by DNA hypermethylation at the promoter-associated CpG islands of the Cby1- encoding gene C22*orf*2. DNA hypermethylation has been involved in BCR ABL1- driven silencing of multiple tumor suppressor genes, eventually associated with the disease progression toward a fully transformed phenotype of BC and/or drug resistance. Its role in Cby1 downmodulation leading to beta catenin activation may be critical in LSC survival and self-renewal.

## Supporting Information

Figure S1 **FISH analyses on interphase nuclei and metaphases of HP. A**: The LSI BCR/ABL DCDF translocation probe lets detect two green signals and two red signals respectively marking BCR on chromosomes 22 and ABL on chromosomes 9 in interphase nuclei of HP. **B**: The C22*orf*2 probe distinguishes two distinct green signals corresponding to the promoter origin and two red signals corresponding to the end of the gene on chromosome 22 in metaphases of HP.
(TIF)

Figure S2 **FISH analyses on variant translocations.** FISH analyses relative to BCR-ABL1 and C22*orf*2 were performed in two additional CML-CP patients not included in the study exhibiting t(7;9;22) (A) and t(1;9;22) (B) variant translocations. As shown in Figure 1, Cby1 signals relocated at the third chromosome involved in translocation.
(TIF)

Figure S3 **Cby1 reduced expression is restricted to the leukemic clone.** The levels of Cby1 protein (upper panel) and transcript (lower panel) in MCF of five CML-CP patients at diagnosis (D) and at the moment of MMR. The results presented have been confirmed in two separate experiments. The vertical line dividing the figure indicates that the results were obtained in two different blots, referred to B2M and actin as internal controls for PCR reaction and protein loading, and compared for signal intensities with HP reference values obtained in the same experiment.
(TIF)

Figure S4 **Prominent reduction of Cby1 expression in the putative LSC compartment.** The levels of Cby1 protein, nuclear beta catenin, Cby1 and cyclin D1 transcripts in MCF and CD34+ cell of HP and six CML-CP patients. See legend to Figure S2 for details.
(TIF)

Table S1 **Clinical details of 40 CML-CP patients included in the study.** The disease prognosis was based on the Sokal score at diagnosis and designated as low, intermediate or high risk of disease progression. Cytogenetic analysis performed at diagnosis underscored the type of translocation. Thirty two patients achieved a complete hematological response (CHR) at the 3^rd^ month of therapy with TK inhibitors. The follow-up of remaining 8 was not sufficient to assess the response to therapy (not evaluable: NE). Thirty one patients achieved a MMR (3 log reduction of BCR-ABL1 transcript levels compared to diagnosis) within the 1^st^ year of therapy. One patient (35) did not achieve a MMR within the same interval and the follow-up of remaining 8 patients was too short to evaluate the molecular response to therapy.
(DOC)

Table S2 **Ratios of Western blot or PCR signal intensities vs HP pool.** Equal amounts of RNA and proteins from MCF of peripheral blood samples of HP (collected after growth factor-induced mobilization from bone marrow and intended for bone marrow transplantation) were pooled to avoid individual differences in transcript and protein expression. PCR and Western blot (WB) signal intensities of the HP pool were normalized to 1 and kept as reference of PCR and Western blot signal intensities of MCF from bone marrow samples of CML-CP patients. ND: not done.
(DOCX)

Table S3 **Ratios of WB and PCR signal intensities of MCF from CML-CP patients at diagnosis and at the moment of MMR vs HP.** CBY1 protein and transcript expression in CML-CP patients at diagnosis (D) and at the moment of MMR was expressed by the ratio between individual Western blot and PCR signal intensities and Western blot and PCR signal intensities of pooled HP normalized to 1.
(DOCX)

Table S4 **Ratios of WB and PCR signal intensities of MCF and CD34+ cells from CML-CP patients vs HP.** CBY1 protein and transcript, beta catenin nuclear protein and cyclin D1 transcript levels in MCF and CD34+ cells of HP and CML-CP patients were expressed as aforesaid.
(DOCX)
