# Supplementary figures and images for: BCR-ABL1-Associated Reduction of Beta Catenin Antagonist Chibby1 in Chronic Myeloid Leukemia
Source: PLoS One. 2013 Dec 10;8(12):e81425. doi: 10.1371/journal.pone.0081425 (PMC3858264; doi:10.1371/journal.pone.0081425)

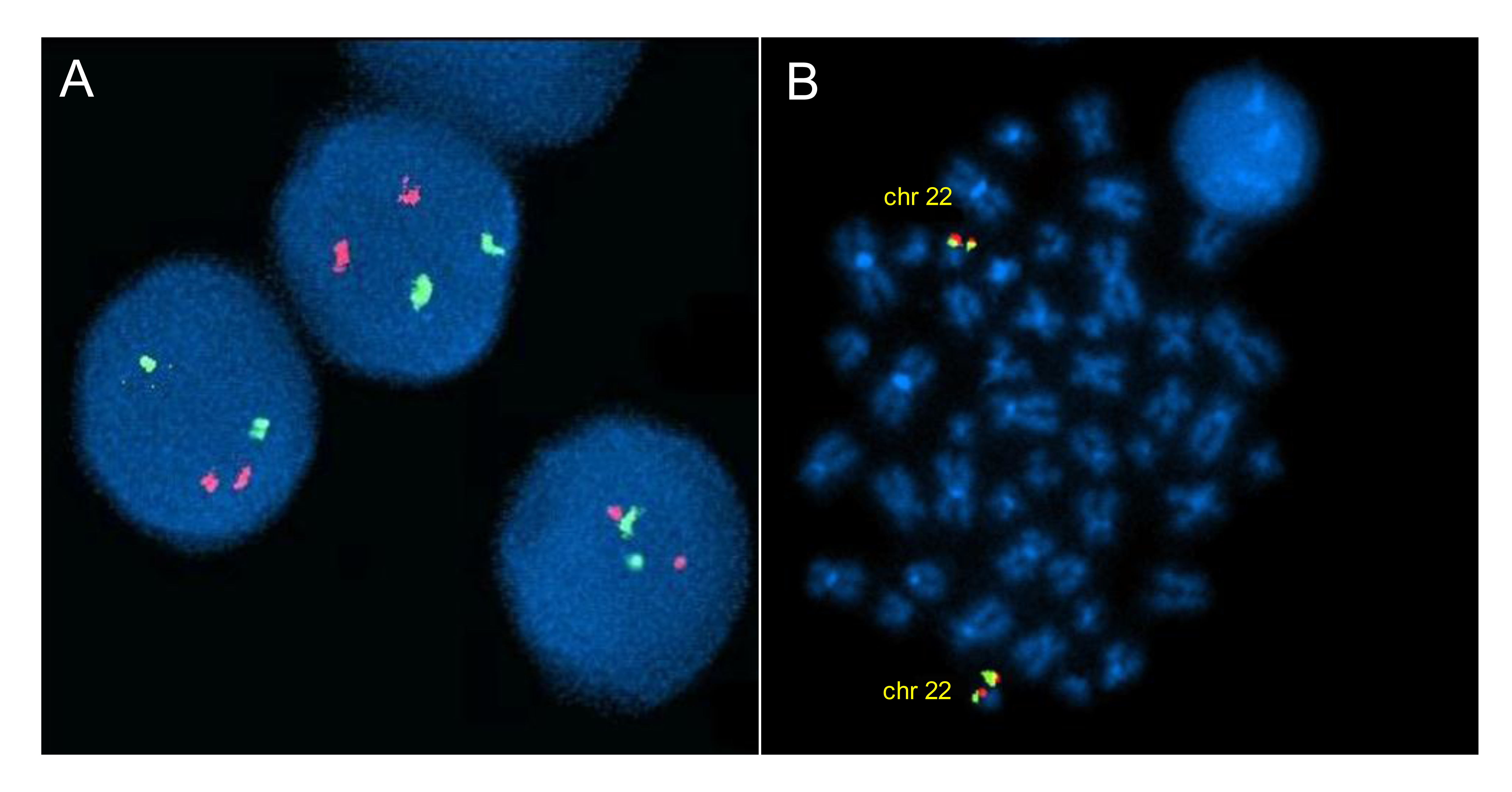

Supplement: Figure S1 — FISH analyses on interphase nuclei and metaphases of HP. A: The LSI BCR/ABL DCDF translocation probe lets detect two green signals and two red signals respectively marking BCR on chromosomes 22 and ABL on chromosomes 9 in interphase nuclei of HP. B: The C22orf2 probe distinguishes two distinct green signals corresponding to the promoter origin and two red signals corresponding to the end of the gene on chromosome 22 in metaphases of HP. (TIF) [file pone.0081425.s001.tif]

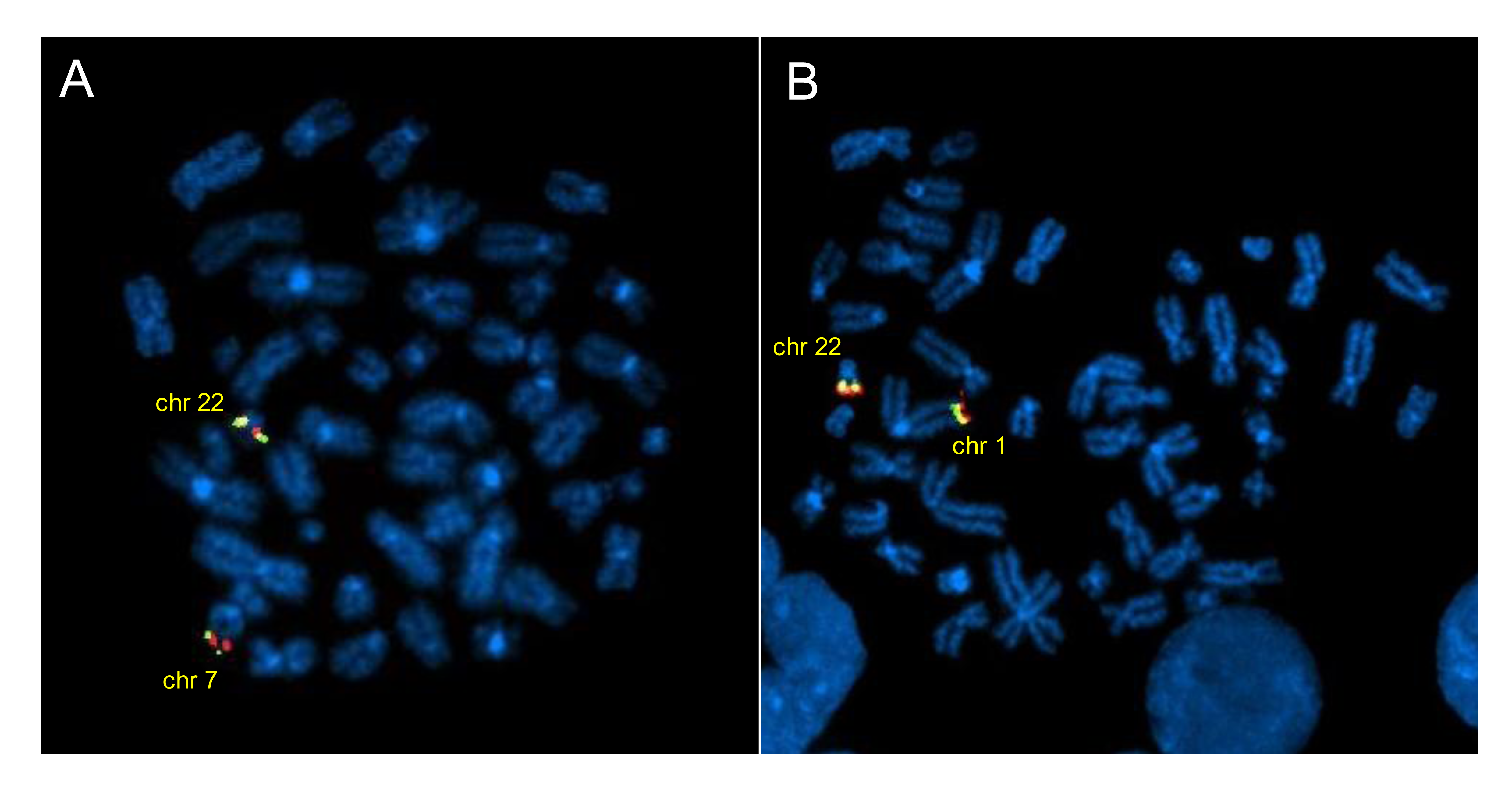

Supplement: Figure S2 — FISH analyses on variant translocations. FISH analyses relative to BCR-ABL1 and C22orf2 were performed in two additional CML-CP patients not included in the study exhibiting t(7;9;22) (A) and t(1;9;22) (B) variant translocations. As shown in Figure 1, Cby1 signals relocated at the third chromosome involved in translocation. (TIF) [file pone.0081425.s002.tif]

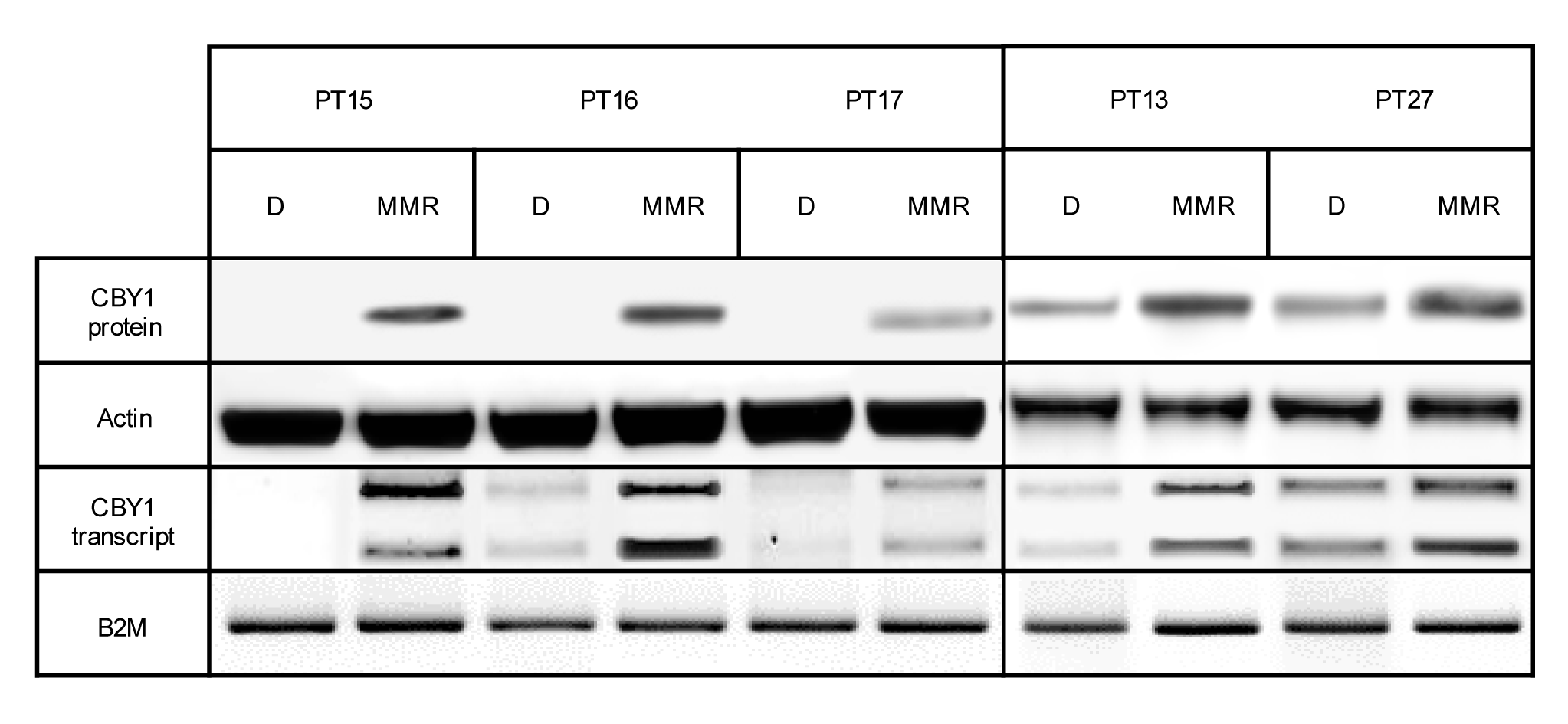

Supplement: Figure S3 — Cby1 reduced expression is restricted to the leukemic clone. The levels of Cby1 protein (upper panel) and transcript (lower panel) in MCF of five CML-CP patients at diagnosis (D) and at the moment of MMR. The results presented have been confirmed in two separate experiments. The vertical line dividing the figure indicates that the results were obtained in two different blots, referred to B2M and actin as internal controls for PCR reaction and protein loading, and compared for signal intensities with HP reference values obtained in the same experiment. (TIF) [file pone.0081425.s003.tif]

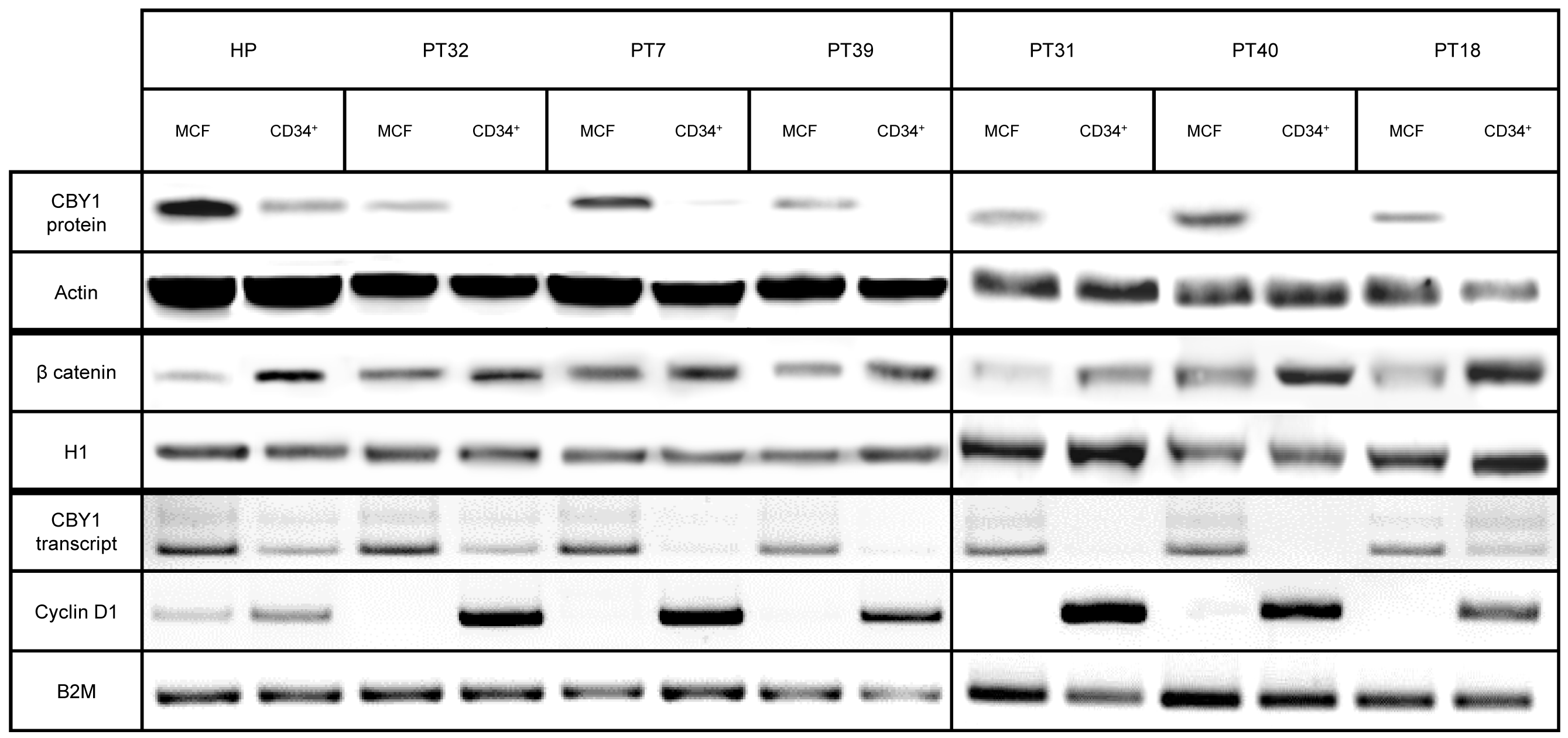

Supplement: Figure S4 — Prominent reduction of Cby1 expression in the putative LSC compartment. The levels of Cby1 protein, nuclear beta catenin, Cby1 and cyclin D1 transcripts in MCF and CD34+ cell of HP and six CML-CP patients. See legend to Figure S2 for details. (TIF) [file pone.0081425.s004.tif]
