# Supplementary material for: BCR-ABL1-Associated Reduction of Beta Catenin Antagonist Chibby1 in Chronic Myeloid Leukemia
Source: PLoS One. 2013 Dec 10;8(12):e81425. doi: 10.1371/journal.pone.0081425 (PMC3858264; doi:10.1371/journal.pone.0081425)
Supplement: Table S1 — Clinical details of 40 CML-CP patients included in the study. The disease prognosis was based on the Sokal score at diagnosis and designated as low, intermediate or high risk of disease progression. Cytogenetic analysis performed at diagnosis underscored the type of translocation. Thirty two patients achieved a complete hematological response (CHR) at the 3rd month of therapy with TK inhibitors. The follow-up of remaining 8 was not sufficient to assess the response to therapy (not evaluable: NE). Thirty one patients achieved a MMR (3 log reduction of BCR-ABL1 transcript levels compared to diagnosis) within the 1st year of therapy. One patient (35) did not achieve a MMR within the same interval and the follow-up of remaining 8 patients was too short to evaluate the molecular response to therapy. (DOC) [file pone.0081425.s005.doc]

**Table S1. Clinical details of 40 CML-CP patients included in the study**

| PATIENT ID | SOKAL SCORE | TRANSLOCATION | CHR | MMR |
| --- | --- | --- | --- | --- |
| 1 | LOW | t (9,22) | YES | YES |
| 2 | LOW | t (9,22) | YES | YES |
| 3 | LOW | t (9,22,17) | YES | YES |
| 4 | LOW | t (9,22) | YES | YES |
| 5 | LOW | t (9,22) | YES | YES |
| 6 | LOW | t (9,22) | YES | YES |
| 7 | LOW | t (9,22) | YES | YES |
| 8 | LOW | t (9,22) | YES | YES |
| 9 | LOW | t (1,9,22) | YES | YES |
| 10 | LOW | t (9,22) | YES | YES |
| 11 | LOW | t (9,22) | YES | YES |
| 12 | LOW | t (9,22) | YES | YES |
| 13 | LOW | t (9,22) | YES | YES |
| 14 | LOW | t (9,22) | YES | YES |
| 15 | LOW | t (9,22) | YES | YES |
| 16 | LOW | t (9,22) | YES | YES |
| 17 | LOW | t (9,22) | YES | YES |
| 18 | LOW | t (9,22) | NE | NE |
| 19 | INTER | t (9,22) | YES | YES |
| 20 | INTER | t (9,22) | YES | YES |
| 21 | INTER | t (9,22) | YES | YES |
| 22 | INTER | t (9,22) | YES | YES |
| 23 | INTER | t (9,22) | YES | YES |
| 24 | INTER | t (9,22) | YES | YES |
| 25 | INTER | t (9,22) | YES | YES |
| 26 | INTER | t (9,22) | YES | YES |
| 27 | INTER | t (6,9,22) | YES | YES |
| 28 | INTER | t (9,22) | YES | YES |
| 29 | INTER | t (9,22) | YES | YES |
| 30 | INTER | t (9,22) | NE | NE |
| 31 | INTER | t (9,22) | NE | NE |
| 32 | HIGH | t (9,22) | YES | YES |
| 33 | HIGH | t (9,22) | YES | YES |
| 34 | HIGH | t (9,22) | YES | YES |
| 35 | HIGH | t (9,22) | YES | NO |
| 36 | HIGH | t (9,22) | NE | NE |
| 37 | HIGH | t (9,22) | NE | NE |
| 38 | HIGH | t (9,22) | NE | NE |
| 39 | HIGH | t (9,22) | NE | NE |
| 40 | HIGH | t (9,22) | NE | NE |

The disease prognosis was based on the Sokal score at diagnosis and designated as low, intermediate or high risk of disease progression. Cytogenetic analysis performed at diagnosis underscored the type of translocation. Thirty two patients achieved a complete hematological response (CHR) at the 3^rd^ month of therapy with TK inhibitors. The follow-up of remaining 8 was not sufficient to assess the response to therapy (not evaluable: NE). Thirty one patients achieved a MMR (3 log reduction of BCR-ABL1 transcript levels compared to diagnosis) within the 1^st^ year of therapy. One patient (35) did not achieve a MMR within the same interval and the follow-up of remaining 8 patients was too short to evaluate the molecular response to therapy.
