# Supplementary material for: BCR-ABL1-Associated Reduction of Beta Catenin Antagonist Chibby1 in Chronic Myeloid Leukemia
Source: PLoS One. 2013 Dec 10;8(12):e81425. doi: 10.1371/journal.pone.0081425 (PMC3858264; doi:10.1371/journal.pone.0081425)
Supplement: Table S2 — Ratios of Western blot or PCR signal intensities vs HP pool. Equal amounts of RNA and proteins from MCF of peripheral blood samples of HP (collected after growth factor-induced mobilization from bone marrow and intended for bone marrow transplantation) were pooled to avoid individual differences in transcript and protein expression. PCR and Western blot (WB) signal intensities of the HP pool were normalized to 1 and kept as reference of PCR and Western blot signal intensities of MCF from bone marrow samples of CML-CP patients. ND: not done. (DOCX) [file pone.0081425.s006.docx]

| PATIENTS | RATIOS OF WB  SIGNAL INTENSITIES  VS HP POOL (=1) | RATIOS OF PCR  SIGNAL INTENSITIES  VS HP POOL (=1) |
| --- | --- | --- |
|  |  |  |
| 1 | 0.015 | 0.662 |
| 2 | 0.380 | 0.771 |
| 3 | 0.000 | 0.927 |
| 4 | 0.008 | 0.685 |
| 5 | 0.005 | 0.814 |
| 6 | 0.000 | 0.166 |
| 7 | 0.885 | 0.840 |
| 8 | 0.214 | 0.890 |
| 9 | 0.210 | ND |
| 10 | 0.937 | 0.642 |
| 11 | 0.923 | 0.646 |
| 12 | 0.000 | 0.747 |
| 13 | 0.458 | 0.213 |
| 14 | 0.000 | ND |
| 15 | 0.000 | 0.105 |
| 16 | 0.000 | 0.356 |
| 17 | 0.000 | 0.214 |
| 18 | 0.221 | 0.876 |
| 19 | 0.021 | 0.834 |
| 20 | 0.517 | 0.175 |
| 21 | 0.083 | 0.635 |
| 22 | 0.801 | 0.448 |
| 23 | 0.000 | 0.896 |
| 24 | 0.970 | 0.770 |
| 25 | 0.000 | 0.763 |
| 26 | 0.000 | 0.842 |
| 27 | 0.645 | 0.789 |
| 28 | 0.198 | ND |
| 29 | 0.000 | 0.802 |
| 30 | 0.254 | 1.020 |
| 31 | 0.394 | 0.773 |
| 32 | 0.118 | 1.065 |
| 33 | 0.000 | 0.117 |
| 34 | 0.658 | 1.150 |
| 35 | 0.631 | 0.690 |
| 36 | 0.954 | 0.932 |
| 37 | 0.000 | 0.852 |
| 38 | 0.188 | 0.798 |
| 39 | 0.225 | 0.611 |
| 40 | 0.498 | 0.927 |

**Table S2. Ratios of Western blot or PCR signal intensities vs HP pool**

Equal amounts of RNA and proteins from MCF of peripheral blood samples of HP (collected after growth factor-induced mobilization from bone marrow and intended for bone marrow transplantation) were pooled to avoid individual differences in transcript and protein expression. PCR and Western blot (WB) signal intensities of the HP pool were normalized to 1 and kept as reference of PCR and Western blot signal intensities of MCF from bone marrow samples of CML-CP patients. ND: not done.
