# Supplementary material for: BCR-ABL1-Associated Reduction of Beta Catenin Antagonist Chibby1 in Chronic Myeloid Leukemia
Source: PLoS One. 2013 Dec 10;8(12):e81425. doi: 10.1371/journal.pone.0081425 (PMC3858264; doi:10.1371/journal.pone.0081425)
Supplement: Table S3 — Ratios of WB and PCR signal intensities of MCF from CML-CP patients at diagnosis and at the moment of MMR vs HP. CBY1 protein and transcript expression in CML-CP patients at diagnosis (D) and at the moment of MMR was expressed by the ratio between individual Western blot and PCR signal intensities and Western blot and PCR signal intensities of pooled HP normalized to 1. (DOCX) [file pone.0081425.s007.docx]

**Table S3**

**Ratios of WB and PCR signal intensities of MCF from CML-CP patients at diagnosis and at the moment of MMR vs HP**

|  | CBY1 PROTEIN | | CBY1 TRANSCRIPT | |
| --- | --- | --- | --- | --- |
| PATIENT | D | MMR | D | MMR |
| 15 | 0.000 | 0.997 | 0.105 | 0.978 |
| 16 | 0.000 | 0.645 | 0.356 | 1.211 |
| 17 | 0.000 | 0.745 | 0.214 | 0.457 |
| 13 | 0.458 | 0.937 | 0.213 | 0.723 |
| 27 | 0.645 | 1.000 | 0.789 | 1.093 |

CBY1 protein and transcript expression in CML-CP patients at diagnosis (D) and at the moment of MMR was expressed by the ratio between individual Western blot and PCR signal intensities and Western blot and PCR signal intensities of pooled HP normalized to 1.
