# Supplementary material for: BCR-ABL1-Associated Reduction of Beta Catenin Antagonist Chibby1 in Chronic Myeloid Leukemia
Source: PLoS One. 2013 Dec 10;8(12):e81425. doi: 10.1371/journal.pone.0081425 (PMC3858264; doi:10.1371/journal.pone.0081425)
Supplement: Table S4 — Ratios of WB and PCR signal intensities of MCF and CD34+ cells from CML-CP patients vs HP. CBY1 protein and transcript, beta catenin nuclear protein and cyclin D1 transcript levels in MCF and CD34+ cells of HP and CML-CP patients were expressed as aforesaid. (DOCX) [file pone.0081425.s008.docx]

**Table S4**

**Ratios of WB and PCR signal intensities of MCF and CD34+ cells from CML-CP patients vs HP.**

|  | CBY1 PROTEIN | | CBY1 TRANSCRIPT | | NUCLEAR B-CATENIN | | CYC D1 TRANSCRIPT | |
| --- | --- | --- | --- | --- | --- | --- | --- | --- |
| PATIENT | MCF | CD34^+^ | MCF | CD34^+^ | MCF | CD34^+^ | MCF | CD34^+^ |
| HP | 1.000 | 0.298 | 1.000 | 0.217 | 1.000 | 3.854 | 1.000 | 2.342 |
| 32 | 0.118 | 0.003 | 1.065 | 0.395 | 2.536 | 3.543 | 0.123 | 6.618 |
| 7 | 0.885 | 0.005 | 0.840 | 0.101 | 2.323 | 3.395 | 0.320 | 6.634 |
| 39 | 0.225 | 0.003 | 0.611 | 0.121 | 2.112 | 3.125 | 0.334 | 3.970 |
| 31 | 0.394 | 0.004 | 0.773 | 0.105 | 1.545 | 3.563 | 0.115 | 7.826 |
| 40 | 0.498 | 0.004 | 0.927 | 0.214 | 3.221 | 6.142 | 0,127 | 6.421 |
| 18 | 0.221 | 0.005 | 0.876 | 0.335 | 2.665 | 6.021 | 0,101 | 4.650 |

CBY1 protein and transcript, beta catenin nuclear protein and cyclin D1 transcript levels in MCF and CD34+ cells of HP and CML-CP patients were expressed as aforesaid.
